# Supplementary material for: Chromothripsis during telomere crisis is independent of NHEJ, and consistent with a replicative origin
Source: Genome Res. 2019 May;29(5):737–49. doi: 10.1101/gr.240705.118 (PMC6499312; doi:10.1101/gr.240705.118)
Supplement: Supplemental Material [file supp_gr.240705.118_Supplemental_file_1.zip › contigs/annotated_contigs/DB57/contig.2.DB57_length_268_mean_cov_4.26492537313.docx]

**DB57_length_268_mean_cov_4.26492537313**

```
CTATTTTCATCAATAGAGCTAATAAATATACGGTACTACTATGTAAAAAGGGCAAACAGCATTACTATGC|CCTTGAT|ATTAGAGACC
 >chr7:153732867-153732944 - E=5e-32                                   >chr7:149761738-
TTGGCAAACGTAAAGTACTTAAAAAATGAATAGACTGCCTTCCGAATGTGAACTCACTGACTAAGCCCAGTGAAAGGAGAGTATGGCAT
149761912 - E=1e-93
GAAATCAGAGAATGATGAATGGCCAGGATTACGTGTGTGTGTGTGTGTGTGTGTGTGTGTGTGTGTGT|GTGTGTGTGTGTGTGTGTGT

GTAA
```
